# Handling multiple testing while interpreting microarrays with the Gene Ontology Database

**DOI:** 10.1186/1471-2105-5-124

**Published:** 2004-09-06

**Authors:** Michael V Osier, Hongyu Zhao, Kei-Hoi Cheung

**Affiliations:** 1 Department of Biological Sciences, Rochester Institute of Technology, 85 Lomb Memorial Drive, Rochester, NY 14623, USA; 2 Dept. of Epidemiology and Public Health, Yale University, New Haven, CT 06520, USA; 3 Dept. of Genetics, Yale University, New Haven, CT 06520, USA; 4 Yale Center for Medical Informatics, 300 George St. Suite 501, New Haven, CT 06511, USA

## Abstract

**Background:**

The development of software tools that analyze microarray data in the context of genetic knowledgebases is being pursued by multiple research groups using different methods. A common problem for many of these tools is how to correct for multiple statistical testing since simple corrections are overly conservative and more sophisticated corrections are currently impractical. A careful study of the nature of the distribution one would expect by chance, such as by a simulation study, may be able to guide the development of an appropriate correction that is not overly time consuming computationally.

**Results:**

We present the results from a preliminary study of the distribution one would expect for analyzing sets of genes extracted from *Drosophila*, *S. cerevisiae*, Wormbase, and Gramene databases using the Gene Ontology Database.

**Conclusions:**

We found that the estimated distribution is not regular and is not predictable outside of a particular set of genes. Permutation-based simulations may be necessary to determine the confidence in results of such analyses.

## Background

With technological improvements and decreasing costs, microarrays are quickly becoming an affordable analytical tool for genetics analysis. Additionally, the arrays being used are of increasing spot density, allowing for more genes to be tested at once. One impact of the resulting increase in data flow is that it will become more likely that a researcher using microarrays will have greater difficulty making sense of results from preliminary statistical analyses without further computational exploration. In other words, once the researcher has received a list of genes, by whatever statistical means, that are differentially expressed, the task of determining the biological implications of that gene list will need to be performed by statistical methods utilizing computers.

Numerous research groups are developing software tools to perform an interpretation of the list of differentially expressed genes, generally by mapping against previously developed knowledgebases such as the Gene Ontology (GO) [1,2] or GenMAPP [3] as a reference data set (reviewed briefly in [4]). Some tools, such as DAVID [5] and FatiGO [6] examine the percentage of the gene list that is directly associated with a node of the knowledgebase. This method is extremely fast due to its simplicity, but it does have disadvantages, which are also due to the simplicity of the analysis. For example, in some of these tools, information about how nodes (biological terms or steps in pathways) of the knowledgebase are related to each other is ignored. Additionally, in hierarchical structures such as GO, genes with a less precise functional definition will be associated with a node closer to the root than a gene with a more precise definition. In such a case, the information content about the two genes is split into different nodes, reducing the power of the analytical method.

Other tools such as GOMiner [7] and MAPPFinder [8] analyze the gene list in a broader context of the knowledgebase, looking for patterns of a larger scale than a single node. MAPPFinder searches for whole pathways (MAPPs) over-represented by the gene list. GOMiner performs analyses using genes associated with a node in GO or genes associated with any children of that node, sometimes called "inclusive analysis". In this way, GOMiner minimizes the power reduction of some simpler methods.

These tools provide a powerful way for the researcher to quickly get a summarization of the gene list within a biological context. One common problem for the inclusive analytical methods, especially those using knowledgebases with polyhierarchical structures (individual nodes can have multiple parents) like GO, is correcting for multiple statistical tests, usually thousands. In such a case, a Bonferroni correction is overly conservative to the point of being counterproductive since few if any results of the interpretation remain significant [7]. As of June, 2003, GODB had >13,000 DAG nodes which may be tested, meaning a correction factor of greater than four orders of magnitude would be needed in a Bonferroni correction. Other standard methods used include controlling the Family-Wise Error Rate (FWER) using a numerical correction of the p-value (discussed in [9]) or controlling the False Positive Rate (FDR, discussed in [10]). In both cases the methodology should again be overly conservative since, when using inclusive analysis, the p-values for each GO term are not independent [11].

Here we present, in the context of the program GOArray [4], a preliminary analysis of the feasibility of using permutation-based simulations to provide an alternate method of handling the multiple-testing problem. GOArray analyzes the gene list in the context of GO. Permutations of the differentially expressed gene list are generated from the total list of genes represented on the microarray to estimate the distribution of significant GO terms expected by chance. We analyze the nature of the distribution of significant terms in reference to varying p-values and numbers of differentially expressed genes using publicly available data sets. We then compare the list of significant terms between data sets. Finally, we discuss the implications of this distribution to provide one solution to the multiple-test problem when analyzing microarray data in the context of GO.

## Results

**Figure 1:**
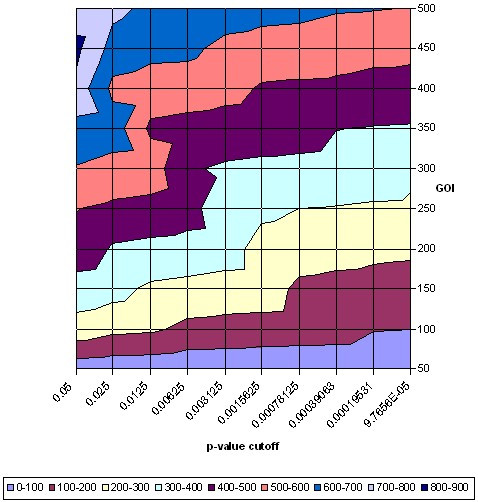
Overhead view of surface topology for the the Arbeitman data set. Different shadings represent different numbers of significant terms.

Four of the test data sets analyzed were extracted from the National Center for Biotechnology Information's (NCBI) Gene Expression Omnibus (GEO) [12]. The first is an array of *Drosophila *markers used by Arbeitman et al. [13] (GEO accession GPL218) for a time-series study of the *Drosophila *life cycle. This array represents 5081 microarray spots, from which there are 2825 genes as represented by unique FlyBase [14] accession numbers. The estimation of the distribution took ~16.6 hours. The mean numbers of significant terms for each combination of p-value cutoff and "Gene of Interest" (GOI) count are presented in Figure 1 (full tables of values for all figures are present in the Additional File 1). A "trough" of less significant terms than the two surrounding GOI counts for the same p-value for term significance can be observed in the topology diagonally from 500 GOI and a p-value of 0.05 down to 250 GOI and a p-value of 0.0031. There are additional, similarly "wave-shaped" features, although of lesser degree. For example, there is one with a slower rate of change running diagonally from 250 GOI in the vicinity of p-values 0.0002 and 0.000098 (285.9 and 283.9 significant terms respectively), and from 500 GOI between p-values 0.0063 and 0.0.0031 (660.4 and 653.3 significant terms respectively). Overall, however, there is an increase in the number of significant terms with both increasing GOI and p-value cutoff. The increase is sharp from 50 to 100 GOI, and then more gradual with increasing GOI. The increase in significant terms with increasing p-value cutoff, however, is much more gradual.

**Figure 2:**
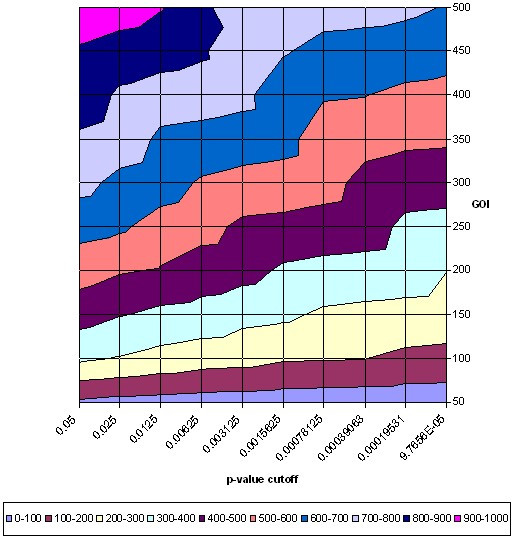
Overhead view of surface topology for the Meiklejohn data set. Different shadings represent different numbers of significant terms.

The second data set is for an array of *Drosophila *markers used by Meiklejohn et al. [15] (GEO accession GPL356) for a study of interspecies variation. The array represents 5928 cDNA probes, from which there are 5375 unique FlyBase accession numbers. The estimation of the distributions took ~23.8 hours. The mean number of significant terms for each combination of p-value and GOI count were estimated by simulation (Figure 2). Again, there is a general increasing trend in the number of significant terms with increasing numbers of GOI and p-value cutoffs. There is another observed trough, however, starting from 500 GOI and a p-value cutoff of 0.0063 diagonally to 350 GOI and a p-value cutoff of 0.00078. As with the first data set, there are also "wave-like" structures in the topology such as from approximately 250 GOI and a p-value of 0.0016 to 400 GOI and a p-value of 0.013. Given the similarity in topology to the first data set, the possibility that these two sets of FlyBase accessions have large overlap was considered. Indeed, ~90% (2560) of the FlyBase accessions from the Arbeitman data set are observed as ~50% of the Meiklejohn data set.

**Figure 3:**
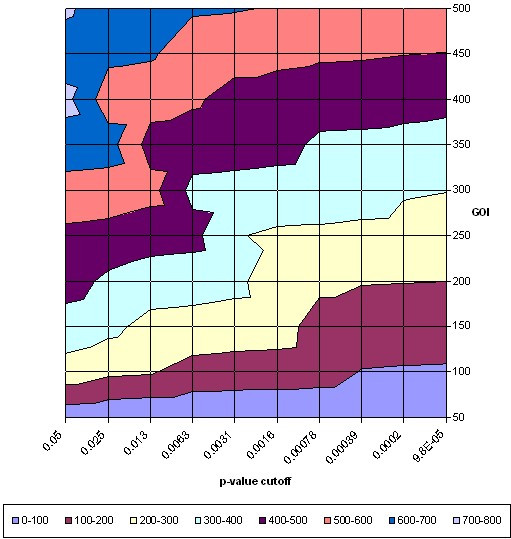
Overhead view of surface topology for the portion of the Meiklejohn data set that did not overlap the Arbeitman data set. Different shadings represent different numbers of significant terms.

That the two data sets would not be independent is to be expected, since one goal of both studies was to examine as many of the known *Drosophila *genes as possible. This non-independence will probably be observed for most pairs of *Drosophila *microarrays. Because of this, we extracted the 2815 FlyBase accession numbers from the Meiklejohn data set that did not overlap with the Arbeitman data set, and estimated the distribution of significant terms for just those genes as a comparison to the other two data sets. The simulation took ~18.3 hours and the results are presented in Figure 3. As with the previous two data sets, there is generally an increase in the number of significant terms with increasing numbers of GOI and p-value cutoffs. There is also another trough extending from 450 and 500 GOI with a p-value cutoff of 0.05 to 200 GOI with a p-value cutoff of 0.0016.

**Figure 4:**
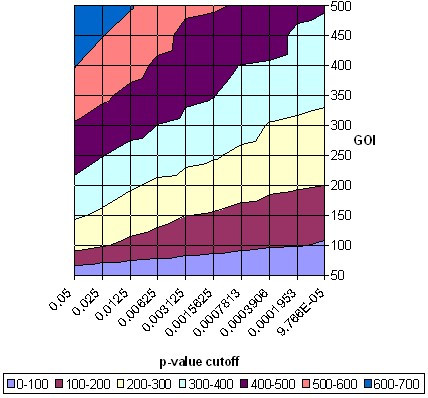
Overhead view of surface topology for the portion of the *S. cerevisiae *data set. Different colors represent different numbers of significant terms.

Since the two real *Drosophila *data sets and one simulated *Drosophila *data set all had a trough in the distribution, it was possible that this is due to inherent structure in GO specifically for *Drosophila*. Therefore, we extracted three other data sets for different species. The first of these non-*Drosophila *sets of genes was for *S. cerevisiae *(GEO accession GPL205), a set of 6084 genes. The overall topology is quite regular (Figure 4). Unlike the *Drosophila *data sets, within the range of GOI and p-values considered there was no evidence of a trough (region where some points would be predicted to have more significant terms than neighboring points but instead have less) in the distribution. There was one data point (500 GOI, p = 0.000391) with a mean number of significant terms (437.3) less than that for the same p-value and the next lower number of GOI (450 GOI, 438.7 terms). The difference between the two means is minute, and may not be meaningful. There are a few regions with leveling (little change in significant terms between points), but these were not large and overall pattern appears somewhat predictable.

**Figure 5:**
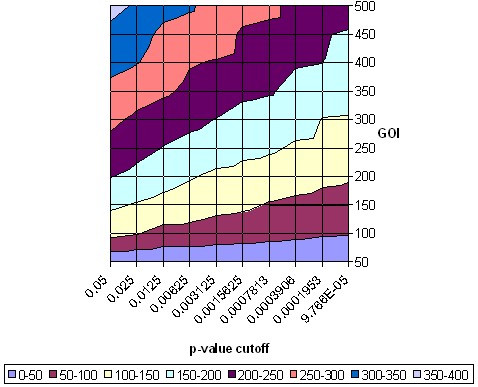
Overhead view of surface topology for the portion of the Wormbase data set. Different colors represent different numbers of significant terms.

**Figure 6:**
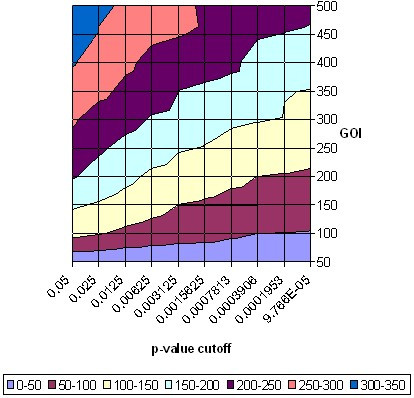
Overhead view of surface topology for the portion of the Gramene data set. Different colors represent different numbers of significant terms.

The second and third non-*Drosophila *data set were constructed by taking all Wormbase [16] and Gramene [17] accessions from GODB. In the case of the Wormbase data set (8224 genes), the distribution again appears somewhat regular (Figure 5), with just a few regions of leveling, but no major trough. There was, however, more "wave-like" structure with increasing numbers of GOI and more stringent p-values. The same was noted for the Gramene data set (4798 genes), although the leveling was considerably more apparent (Figure 6). This was especially true in the region from 500 GOI and p = 0.00156 to 300 GOI and p = 9.8 × 10^-5^. Even with this region, however, the distribution appears somewhat smoother than that observed for the *Drosophila *data set. The region in question is located near the edge of the explored space, however, and a pattern may emerge with higher number of GOI.

**Figure 7:**
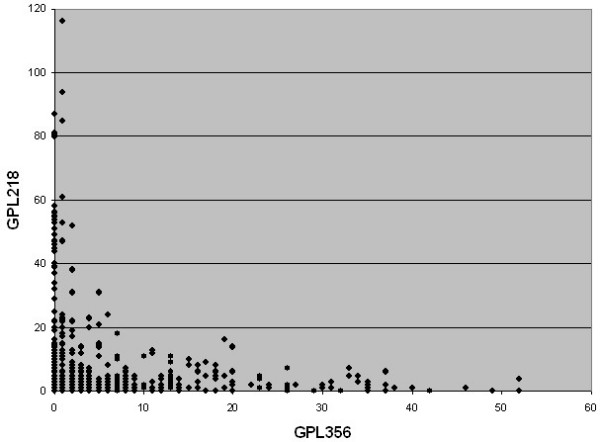
Correlation in significant terms between data sets. The number of times each term was observed as significant based on the analysis of two different sets of *Drosophila *genes are scatter plotted.

Finally, to see if the same terms were consistently appearing as significant in the *Drosophila *data sets, we compared the actual number of significant occurrences for each term for the two data sets extracted from GEO (GPL218 and GPL356). Genes with a five-fold or greater change in expression were chosen as GOI. A p-value cutoff of 0.001 was chosen. The list of terms that came up as significant, and the number of permutations out of 1000 that were significant, was recorded and presented as a scatter plot (Figure 7). From the plot, it can clearly be seen that there is a lack of correlation between the number of times a term appears as a significant in one data set compared to the second data set, even accounting for a different maximum number of significant terms in the two data sets. A handful of terms were significant a similar number of times in each data set relative to the maximum count of significant terms for the respective data sets. In other words, a handful of terms mapped near the line extending from the origin to the point marked by the maximum value along each axis, which would mark roughly equivalent relative occurrences of the term as significant between the two data sets. However, these terms were near the origin and the vast majority of points were along the axes, showing a clear lack of correlation in how often terms were observed as significant between these two closely related lists of genes.

## Discussion

Based on this set of simulations, predictability appears to be limited to specific data sets. One method of correcting our expectations after performing multiple tests would be to calculate a factor by which to modify α based on the DAG of GO terms. In other words, one could use an adjusted p-value to control the FWER or FDR. Controlling for these two types of error by use of adjusted p-values, however, assumes independence of the tests [11]. Since there is currently no practical method for directly untangling the interdependence of terms in the GO hierarchy to generate a less conservative correction, adjusted p-values are limited to overly strict results.

Another method would be to determine a formula that conservatively approximates the simulated distribution. Unfortunately, the only commonality between the distributions is that, with the exception of the *Drosophila *data sets, the number of significant terms increases with an increasing number of GOI and an increasing p-value cutoff. The magnitude and detailed shape of the distribution varies between all tested data sets. Even in the more regular non-*Drosophila *data sets, there were some fluctuations in the distribution, and a smooth surface was not observed. Since neither of the two methods of correcting expectations is currently feasible, it appears that, for now, we are forced to rely on simulation-based methods to estimate the expected distribution of significant terms for each set of genes being examined.

While it would be desirable to have a smooth topology that allows for a simple formulaic calculation of the number of significant terms one would expect by chance, it is unfortunately not observed for the *Drosophila *data sets examined here. The trough that disrupts the *Drosophila *data sets was not observed, however, in the data sets for other species. The cause of this trough is undetermined, but may be due to structure within the graph of GO terms associated with FlyBase accessions. Alternatively, there could be structure within the chosen genes that is more evident with smaller data sets, since the trough appears to be deepest for the two smaller data sets. One way to approach the question of cause would be to examine which, if any, terms are observed disproportionately in the permuted sets. Based on the frequency of terms it may be possible to observe a pattern in either the genes tested or the set of associated GO terms. We have been unable to observe such a pattern, but that does not mean it does not exist. If one could be found, it may give insights into how to dissect the structure, possibly leading to a more elegant solution to the multiple test problem than a simulation-based approach.

Though we were unable to find hints of an easy formulaic way to correct our expectations, we may be able to find a practical (e.g., efficient) method of correction through simulations. There are several ways in which simulated estimates of the distribution could be implemented to provide a less conservative method, yet still statistically appropriate, than a Bonferroni correction to handle the problem of correcting our expectations after performing multiple statistical tests. The simplest to implement, and likely the most accurate, would be to perform a permutation-based simulation for each analysis of a microarray data set in the context of GO. The primary problem with this approach is that it is computationally intensive since the GOI would need to be permutated and scored a thousand or more times for every analysis of a microarray. While tools such as parallel processing can reduce the absolute time necessary to perform the simulations, it is not the most elegant way to solve the problem.

Another method would be to simply generate the estimated distribution, again using a permutation-based simulation, once for each set of accession numbers (e.g., each microarray design) for a range of GOI counts and p-value cutoffs, similar to what we have done here but in finer detail, and storing the results. The most conservative simulation distribution neighboring the experimental combination of p-value and GOI count could then be extracted from the stored table to provide an estimated distribution. One problem with this approach is determining how fine a table to design (e.g., the number of values to simulate for each of the two primary parameters). With a simple 10 × 10 matrix, the simulation took ~16–24 hours on a single 2.4 GHz Xeon processor. A finer matrix of parameter values will result in a better estimation of the topology, but consumes more time to compute in a non-linear fashion. However, if a large number of microarray experiments is to be conducted with a single geometry, this method would reduce the total time to estimate significance across all experiments since the simulation would only need to be performed once. Additionally, it will be necessary to determine what range of values should be considered. For the smallest data set tested here (>2500 FlyBase accessions), GOI lists representing less than 20% (500) of the accession numbers were used. The amount of computation time that should be dedicated to simulating the distribution of significant terms expected by chance will likely be a balance determined by the computing resources available, estimates of how many experiments will use the array design, and minimal p-value cutoffs and maximal GOI parameter values determined by the predicted user needs.

## Conclusions

Based on the large simulations performed here, it appears that the rate at which terms are observed as significant is not predictable between sets of genes for a given GOI count and p-value cutoff. Even within a particular species, there is no correlation in relative frequency at which particular terms are significant. Therefore, permutation-based simulations appear to be the most reliable way to generate an estimate of the expected distribution of significant terms. As a result, we plan to extend the confidence tests in the next version of GOArray (version 2.0) by implementing a "false positive frequency estimation" for individual terms based on simulation results. Also, since which terms are observed as significant appears to be highly dependent on the structure of the gene list, and possibly the list of GOI, we plan to examine the merits of bootstrap methods (e.g. in the simulations choosing GOI from the original list of GOI with replacement) rather than a strict permutation method (e.g. choosing GOI from the total list of genes without replacement).

In the best case, it appears feasible to pre-generate the estimated distribution of the number of significant terms through a permutation-based simulation, then use a lookup table during analyses of experimental data sets. In the worst case, one would need to generate the distribution for each experimental data set, possibly testing various p-value cutoffs to determine where power is maximal. Even in the worst case, currently available processing power allows the test for a single set of genes and a single p-value cutoff to be performed in well under an hour. While near-instant results would be desirable by end users, the worst case scenario is still quite practical and will only improve over time alongside general computer performance. Thus, relying on permutation-based methods may not be a serious inconvenience, and in fact a highly accurate method of assessing our confidence in the results of the analysis.

## Methods

### Test System

All tests were performed on a single processor of a dual Xeon 2.4 MHz CPU system with 2 gigabytes of RAM. The operating system was RedHat Linux 7.3 with an SMP kernel. All time calculations were determined using the Linux command *time*.

### GOArray

GOArray is a Perl script that maps genes of interest (GOI) and non-GOI (NGOI), where the difference between the two gene lists is determined by the researcher, from a microarray experiment to terms in GO and all of that term's parent terms. The GO rooted-DAG is represented in a hash table using the GODB field terms.id as the keys. A z-score is assigned to each term based on the number of genes associated with that term or any of its children relative to the total numbers of GOI and NGOI. Z-scores were used to calculate p-values since they are easy and efficient to compute, and they approximate the hypergeometric p-values when the number of NGOI and GOI for the entire data set is large compared to the NGOI and GOI for the individual nodes. Terms with only one gene in the numerator (GOI) are not given a z-score since it is not possible to have an overrepresentation of GOI with a single gene. P-values are determined using twice the value (e.g., a two-sided test) returned by the routine "uprob($z)" (where "$z" is the z-score) from the Perl module Statistics::Distributions available from the Comprehensive Perl Archive Network (CPAN) [18]. The June 2003 GODB data set is used in this analysis.

Simulations of the GOI list are performed by permuting the status of each gene, keeping the total number of GOI constant. For example, in the case of an experiment examining 5000 total genes with 100 GOI, in each simulation 100 of the 5000 genes are assigned the status of GOI, and 4900 genes are assigned the status of NGOI.

The only modifications to the GOArray source code in this analysis are the addition of loop structures to iterate the numbers of GOI and count the number of significant terms under different p-value cutoffs to determine when a term is significant, the use of a user-determined random number seed rather than a computer determined one for reproducibility, and the addition of a routine to summarize the simulation data.

The source code for both GOArray and the modifications discussed here are available on the Web [19].

### Distributions

Using the modified GOArray code, the number of significant terms were determined for p-values (determining which terms were significant, not which genes were GOI) from 0.05 down to ~0.000098 (starting with 0.05 and decreasing the p-value by a factor of 2 with each iteration), and GOI counts from 50 to 500 in increments of 50. This generates the number of significant terms for each of 1000 permutations for all combinations of ten different p-values and ten different GOI counts, for a total of 100 distributions of 1000 permutations for each data set.

## Authors' Contributions

MVO conceived of the study, performed the analyses, and drafted the manuscript. HYZ participated in the statistical design. KHC participated in the study design and coordination.

## Supplementary Material

Additional File 1 A Microsoft Word document containing the data tables used to generate Figures 1 through 6. Click here for file
