# Supplementary material for: Handling multiple testing while interpreting microarrays with the Gene Ontology Database
Source: BMC Bioinformatics. 2004 Sep 6;5:124. doi: 10.1186/1471-2105-5-124 (PMC518975; doi:10.1186/1471-2105-5-124)
Supplement: Additional File 1 — A Microsoft Word document containing the data tables used to generate Figures 1 through 6. [file 1471-2105-5-124-S1.doc]

**Tables**

**Table 1.** Mean number of significant terms for each combination of p-value cutoff and GOI count for the Arbeitman data set. P-value cutoffs are rounded to two digits in the table (see methods).

|  | **p-value cutoff** | | | | | | | | | |
| --- | --- | --- | --- | --- | --- | --- | --- | --- | --- | --- |
| **GOI** | **0.05** | **0.025** | **0.013** | **0.0063** | **0.0031** | **0.0016** | **0.00078** | **0.00039** | **0.00020** | **0.000098** |
| **50** | 39.1 | 33.7 | 27.5 | 24.3 | 22.0 | 19.4 | 17.3 | 14.9 | 14.5 | 12.5 |
| **100** | 263.5 | 227.3 | 215.8 | 176.5 | 168.7 | 162.7 | 160.3 | 154.3 | 104.9 | 100.0 |
| **150** | 351.6 | 337.2 | 281.9 | 269.2 | 255.1 | 250.9 | 178.9 | 168.1 | 165.9 | 161.7 |
| **200** | 468.2 | 386.9 | 371.2 | 362.0 | 354.4 | 251.1 | 242.4 | 239.1 | 221.0 | 216.3 |
| **250** | 503.0 | 481.7 | 472.1 | 445.0 | 331.0 | 326.8 | 299.6 | 295.0 | 285.9 | 283.9 |
| **300** | 593.9 | 561.1 | 552.9 | 410.8 | 387.2 | 378.6 | 368.3 | 362.4 | 357.8 | 325.1 |
| **350** | 671.2 | 655.5 | 482.1 | 465.6 | 456.9 | 451.7 | 448.8 | 401.7 | 394.7 | 391.5 |
| **400** | 770.0 | 573.6 | 560.7 | 553.5 | 533.5 | 489.2 | 483.1 | 480.6 | 468.8 | 455.7 |
| **450** | 829.0 | 660.2 | 625.9 | 622.8 | 572.2 | 563.5 | 557.3 | 540.3 | 530.2 | 530.1 |
| **500** | 736.6 | 726.7 | 675.1 | 660.4 | 653.3 | 630.4 | 624.6 | 609.7 | 605.4 | 596.7 |

**Table 2.** Mean number of significant terms for each combination of p-value cutoff and GOI count for the Meiklejohn data set. P-value cutoffs are rounded to two digits in the table (see methods).

|  | **p-value cutoff** | | | | | | | | | |
| --- | --- | --- | --- | --- | --- | --- | --- | --- | --- | --- |
| **GOI** | **0.05** | **0.025** | **0.013** | **0.0063** | **0.0031** | **0.0016** | **0.00078** | **0.00039** | **0.00020** | **0.000098** |
| **50** | 77.5 | 69.3 | 63.0 | 57.9 | 53.1 | 48.5 | 46.5 | 43.4 | 41.0 | 39.6 |
| **100** | 319.4 | 293.1 | 265.4 | 246.0 | 237.6 | 211.9 | 207.2 | 202.2 | 175.9 | 170.1 |
| **150** | 441.9 | 407.0 | 376.9 | 365.7 | 328.7 | 318.0 | 284.3 | 273.4 | 264.0 | 259.4 |
| **200** | 547.2 | 509.5 | 492.2 | 447.1 | 438.5 | 385.2 | 370.3 | 363.6 | 353.7 | 302.0 |
| **250** | 631.6 | 617.4 | 557.9 | 538.7 | 477.2 | 469.7 | 457.0 | 447.7 | 376.5 | 371.8 |
| **300** | 735.7 | 670.8 | 650.1 | 588.1 | 569.1 | 557.5 | 541.1 | 467.2 | 447.8 | 441.9 |
| **350** | 784.2 | 757.9 | 680.7 | 669.3 | 649.1 | 636.2 | 540.3 | 532.7 | 520.4 | 514.8 |
| **400** | 864.5 | 782.3 | 754.4 | 742.8 | 730.3 | 626.8 | 611.6 | 604.2 | 576.1 | 568.0 |
| **450** | 890.5 | 861.6 | 847.6 | 817.6 | 718.9 | 709.3 | 672.5 | 664.5 | 660.1 | 643.0 |
| **500** | 955.2 | 943.0 | 906.9 | 806.0 | 784.3 | 756.8 | 734.7 | 730.0 | 718.2 | 694.7 |

**Table 3.** Mean number of significant terms for each combination of p-value cutoff and GOI count for the portion of the Meiklejohn data set that did not overlap the Arbeitman data set. P-value cutoffs are rounded to two digits in the table (see methods).

|  | **p-value cutoff** | | | | | | | | | |
| --- | --- | --- | --- | --- | --- | --- | --- | --- | --- | --- |
| **GOI** | **0.05** | **0.025** | **0.013** | **0.0063** | **0.0031** | **0.0016** | **0.00078** | **0.00039** | **0.00020** | **0.000098** |
| **50** | 28.0 | 22.1 | 17.7 | 13.7 | 11.5 | 9.2 | 8.7 | 7.7 | 5.8 | 5.4 |
| **100** | 265 | 217.7 | 208.4 | 163.6 | 158.1 | 155.7 | 148.4 | 96.1 | 90.8 | 89.7 |
| **150** | 347.2 | 327.8 | 267.4 | 261.3 | 248.2 | 243.6 | 158.0 | 156.0 | 151.7 | 143.5 |
| **200** | 448.8 | 378.0 | 352.3 | 344.7 | 331.3 | 227.3 | 222.6 | 204.6 | 202.7 | 200.5 |
| **250** | 476.8 | 466.8 | 439.4 | 431.8 | 306.5 | 284.9 | 282.0 | 274.1 | 271.9 | 266.7 |
| **300** | 560.4 | 552.8 | 534.4 | 375.0 | 367.9 | 357.2 | 351.9 | 346.8 | 307.8 | 301.5 |
| **350** | 656.4 | 644.6 | 459.7 | 449.6 | 441.3 | 436.0 | 381.1 | 378.1 | 374.5 | 365.1 |
| **400** | 727.8 | 549.4 | 542.9 | 514.1 | 467.2 | 464.5 | 446.4 | 443.5 | 429.5 | 423.8 |
| **450** | 651.1 | 622.1 | 611.3 | 553.3 | 538.7 | 520.4 | 513.0 | 509.0 | 501.7 | 498.7 |
| **500** | 717.0 | 643.5 | 634.5 | 610.8 | 606.1 | 594.5 | 587.9 | 581.0 | 577.2 | 525.9 |

**Table 4.** Mean number of significant terms for each combination of p-value cutoff and GOI count for the portion of the *S. cerevisiae* data set. P-value cutoffs are rounded to two digits in the table (see methods).

| **GOI** | **0.05** | **0.025** | **0.013** | **0.0063** | **0.0031** | **0.0016** | **0.00078** | **0.00039** | **0.00020** | **0.000098** |
| --- | --- | --- | --- | --- | --- | --- | --- | --- | --- | --- |
| **50** | 30.3 | 24.3 | 20.2 | 16.3 | 13.6 | 11.8 | 10.1 | 9.0 | 7.9 | 6.8 |
| **100** | 236.2 | 207.6 | 179.6 | 166.0 | 148.0 | 134.9 | 120.9 | 106.6 | 104.4 | 92.1 |
| **150** | 311.5 | 285.4 | 247.8 | 224.7 | 201.3 | 193.6 | 173.3 | 168.8 | 150.4 | 147.2 |
| **200** | 381.1 | 342.6 | 311.4 | 281.7 | 272.8 | 246.0 | 237.5 | 214.3 | 207.3 | 200.7 |
| **250** | 441.3 | 403.2 | 366.0 | 352.7 | 318.8 | 309.6 | 279.7 | 269.5 | 233.0 | 227.3 |
| **300** | 493.7 | 454.2 | 435.9 | 397.6 | 382.1 | 346.5 | 333.1 | 295.0 | 284.1 | 272.4 |
| **350** | 544.5 | 517.6 | 473.9 | 456.4 | 411.6 | 403.3 | 351.1 | 336.7 | 329.9 | 319.4 |
| **400** | 604.4 | 552.9 | 534.7 | 482.7 | 466.6 | 415.9 | 397.8 | 391.1 | 377.8 | 330.9 |
| **450** | 628.6 | 602.8 | 557.8 | 535.0 | 473.7 | 462.2 | 444.3 | 438.7 | 380.0 | 373.0 |
| **500** | 683.1 | 622.0 | 607.8 | 541.6 | 520.0 | 511.1 | 489.5 | 437.3 | 428.0 | 408.6 |

**Table 5.** Mean number of significant terms for each combination of p-value cutoff and GOI count for the portion of the Wormbase data set. P-value cutoffs are rounded to two digits in the table (see methods).

| **GOI** | **0.05** | **0.025** | **0.013** | **0.0063** | **0.0031** | **0.0016** | **0.00078** | **0.00039** | **0.00020** | **0.000098** |
| --- | --- | --- | --- | --- | --- | --- | --- | --- | --- | --- |
| **50** | 14.2 | 10.4 | 8.7 | 6.8 | 5.9 | 5.2 | 4.5 | 3.9 | 3.6 | 3.0 |
| **100** | 114.0 | 102.5 | 83.2 | 85.3 | 79.4 | 73.7 | 68.7 | 62.1 | 56.6 | 54.7 |
| **150** | 159.7 | 146.2 | 135.0 | 122.3 | 112.1 | 108.5 | 96.9 | 89.0 | 85.8 | 77.0 |
| **200** | 201.7 | 187.7 | 169.0 | 154.2 | 139.9 | 136.4 | 123.6 | 120.9 | 109.3 | 106.3 |
| **250** | 234.8 | 215.0 | 198.0 | 183.3 | 175.3 | 160.7 | 157.4 | 142.8 | 139.6 | 121.2 |
| **300** | 260.6 | 241.3 | 223.4 | 213.5 | 198.5 | 179.1 | 175.2 | 170.3 | 148.4 | 146.0 |
| **350** | 290.6 | 267.7 | 258.1 | 238.3 | 219.6 | 211.5 | 205.0 | 183.3 | 177.1 | 172.4 |
| **400** | 309.7 | 302.1 | 279.2 | 253.4 | 246.9 | 239.1 | 213.4 | 204.0 | 200.7 | 174.6 |
| **450** | 341.9 | 317.4 | 288.8 | 279.7 | 273.1 | 242.9 | 235.2 | 231.3 | 202.6 | 195.5 |
| **500** | 360.0 | 346.4 | 317.1 | 306.4 | 275.9 | 269.6 | 263.6 | 231.5 | 224.9 | 220.9 |

**Table 6.** Mean number of significant terms for each combination of p-value cutoff and GOI count for the portion of the Gramene data set. P-value cutoffs are rounded to two digits in the table (see methods).

| **GOI** | **0.05** | **0.025** | **0.013** | **0.0063** | **0.0031** | **0.0016** | **0.00078** | **0.00039** | **0.00020** | **0.000098** |
| --- | --- | --- | --- | --- | --- | --- | --- | --- | --- | --- |
| **50** | 15.6 | 12.5 | 10.5 | 8.2 | 6.8 | 6.0 | 5.3 | 4.6 | 3.7 | 3.3 |
| **100** | 114.0 | 103.3 | 90.3 | 81.1 | 72.7 | 69.4 | 58.8 | 50.4 | 49.3 | 47.8 |
| **150** | 157.2 | 145.4 | 130.7 | 117.0 | 99.8 | 94.6 | 81.2 | 78.0 | 76.3 | 66.9 |
| **200** | 204.0 | 181.7 | 162.2 | 140.8 | 132.3 | 117.4 | 114.1 | 99.5 | 97.4 | 92.8 |
| **250** | 230.7 | 207.6 | 183.1 | 172.8 | 153.1 | 148.2 | 132.1 | 126.4 | 121.9 | 119.8 |
| **300** | 256.9 | 225.4 | 217.6 | 195.4 | 188.0 | 164.6 | 158.1 | 152.5 | 148.1 | 126.6 |
| **350** | 274.1 | 263.9 | 235.8 | 224.2 | 199.9 | 192.2 | 185.0 | 180.3 | 151.3 | 148.4 |
| **400** | 304.1 | 276.4 | 261.5 | 234.5 | 225.9 | 218.5 | 209.1 | 182.1 | 176.5 | 171.1 |
| **450** | 312.3 | 298.8 | 271.6 | 259.8 | 253.1 | 241.4 | 211.5 | 204.2 | 198.9 | 194.5 |
| **500** | 339.2 | 303.2 | 297.6 | 283.2 | 275.8 | 236.1 | 233.4 | 227.6 | 218.0 | 211.5 |
